# Genetics of Malignant Hyperthermia

**DOI:** 10.1100/tsw.2006.289

**Published:** 2006-12-28

**Authors:** Barbara W. Brandom

**Affiliations:** ^1^ Department of Anesthesiology, University of Pittsburgh Medical Center, USA; ^2^ Children's Hospital of Pittsburgh, USA; ^3^ North American Malignant Hyperthermia Registry of MHAUS (Malignant Hyperthermia Assoc. of the United States), USA

**Keywords:** malignant hyperthermia, ryanodine receptor type one

## Abstract

Study of the genetics of the malignant hyperthermia syndrome began in families in which both malignant hyperthermia (MH) episodes had been experienced and individuals had strongly positive contracture tests diagnostic of susceptibility to MH. Linkage studies associated this MH phenotype to the ryanodine receptor gene (*RYR1*) at chromosome 19q13.1 in many families. Although the MH phenotype is not always linked to chromosome 19, the *RYR1* has remained the focus of experimentation. Other candidate genes exist, but few MH-susceptible families have variants of these genes. Hundreds of MH-susceptible people have variants of *RYR1*.

## BACKGROUND

Malignant hyperthermia (MH) is a pharmacogenetic syndrome. MH was first recognized during general anesthesia with potent inhalation agents because increasing metabolism of skeletal muscle became life threatening. It is known that elevated intracellular calcium in skeletal muscle is the cause of increased metabolism in the MH syndrome. Calcium transport from the sarcoplasmic reticulum of muscle through the ryanodine receptor into the sarcoplasm occurs during the excitation and contraction of muscle. Therefore the ryanodine receptor gene (*RYR1*) is a likely candidate gene for MH. Since the beginning of research into the pathophysiology of this syndrome, the pig has been a useful model. MH episodes can be produced in pigs. The bioassay of muscle developed to identify MH-susceptible individuals, the caffeine halothane contracture tests (CHCT), is highly reproducible in pigs. In five breeds of pig, the Arg615Cys variant in the ryanodine receptor type one protein (RYR1) is linked to MH susceptibility. Therefore, linkage between MH susceptibility in humans and the *RYR1* was examined and established in many families[1,2]. The *RYR1* at 19q13.1 became locus MHS1.

## RYANODINE RECEPTOR TYPE ONE

MH Diagnostic Centers in Europe (see www.emhg.org) have accumulated significant experience with screening of *RYR1* in MH-susceptible (MHS) families. This led to clinical diagnostic testing of MH susceptibility based on *RYR1* mutations. For example, in a family known to have MHS in which a MH-

causative *RYR1* mutation has been identified, it is recommended that first-degree relatives undergo genetic testing for that mutation prior to muscle contracture testing[3]. This strategy leads to identification of the familial mutation in ~50% of family members, as expected for an autosomal dominant condition[4]. Increasing numbers of people from known MHS families have the diagnosis of MHS confirmed in this manner and thus avoid muscle contracture testing. When muscle contracture testing is impossible, such as in newborns[5], MH status can be evaluated with this blood-based test. However, if the familial variant is not found, it is still possible that the individual is MHS. The genetic variants that are associated with MH have not been completely described. Two MH-causative mutations have been observed in rare individuals[6]. In 2006, only a negative contracture test can confirm the diagnosis of "not MH susceptible". As more patients with MHS diagnosed by muscle contracture testing undergo genetic study, the negative predictive value of genetic testing should increase. The addresses of centers where the specialized muscle contracture test, the CHCT, can be performed in North America are listed at www.mhaus.org. The European Centers, which perform IVCT (IVCT is the muscle contracture test performed by the European MH Group with the definition that MHS is present when a contracture of >0.2 g is present at or below a concentration of 2 m*M* caffeine and at or below 2% halothane), are listed at www.emhg.org. This form of the muscle bioassay is also performed in New Zealand.

The size of the *RYR1*, 106 exons[7], which encodes the homotetrameric ryanodine receptor channel (RYR1), led early investigators to focus on the mutation hot spots rather than the entire gene in their search for MH-causative mutations. Many MH-causative mutations were found in hot spot one, the N-terminus (MH1), and in hot spot two (MH2). MH1 includes exons 2 through 17. MH2, the central hot spot, includes exons 39 through 46. MH3, the C-terminus third hot spot, includes exons 90 through 106. Often studies of *RYR1* genetics in different populations examined a limited number of different exons in these hot spots. For example, in Leipzig, Germany, *RYR1* exons 2, 6, 9, 11, 12, 14, 15, 17, 39, 40, 45, 46, and 102 were examined by direct sequencing in 56 MHS people[8]. This was the first study in which mutations in *RYR1* were found in ~70% of the individuals examined. In 124 North American MHS patients, exons 44, 95, 100, and 101 were examined in addition to those studied in Leipzig, but exons 14 and 15 were not examined. A total of 14 *RYR1* variants were found in 23% of these people in North America[9]. Other studies found mutation frequencies in between these numbers. For example, 40% of 48 MHS Swiss had one of the 23 *RYR1* mutations acknowledged by the European Malignant Hyperthermia Group (EMHG) as causative for this syndrome[10]. In 500 unrelated European MHS people, one of 15 MH-causative *RYR1* mutations was found in ~30%[11]. These studies may underestimate the frequency of *RYR1* variants in MHS people because only part of *RYR1* was examined.

Particular mutations in *RYR1* are more common in people from different geographic areas.(see Table 1). In Leipzig, the mutation in exon 17 of *RYR1*, which produces a substitution of cysteine for arginine in the RYR1 protein, referred to as Arg614Cys, was the most frequently observed mutation, followed by Thr2206Met in exon 39 and Arg2454His in exon 46. Together these three mutations were found in 45% of the 56 MHS people studied[8]. In Switzerland, 18% of 62 MHS families had one of three *RYR1* mutations, Arg614Cys, Val2168Met in exon 39, or Gly2434Arg in exon 45[4]. In England, Gly2434Arg and Val2168Met are common *RYR1* mutations[12]. In France, other parts of Germany, and Italy, Arg614Cys was also frequent. Gly341Arg in exon 11 was common in Belgium, France, and the U.K.[11,13]. The most frequent *RYR1* mutation in the U.K., Gly2434Arg, was present, but not the most common, in other European countries[11] (see Table 1). In Europe, just over half of the *RYR1* mutations were found in MH1[13]. However, in 50 Italian subjects, *RYR1* mutations were more frequent in MH2 than in MH1[14]. In the North American study, 70% of the mutations were in MH2. The most frequent mutation in the North American population, Gly2434Arg, was found in only 5% of 124 subjects. Arg2454His and Arg614Cys were the next most common *RYR1* variants in North America[9]. These were not the most common *RYR1* mutations in a group of Japanese MHS people[15]. Therefore, clinical tests of MHS based on the genetics of *RYR1* may elect to examine exons in a sequential fashion based on the frequency of mutations in that population. This will result in a diagnosis of MHS with less expense, but such a strategy can never by itself be adequate to support optimal understanding of the genetics of the MH syndrome.

**TABLE 1**
**Geographic Variability in Common *RYR1* Variants***

| | Gly341Arg | Arg614Cys | Val2168Met | Thr2206Met | Gly2434Arg |
|---|---|---|---|---|---|
| Leipzig, Germany | 5 | **31** | 3 | **23** | 5 |
| Switzerland | 0 | 13 | **69** | 0 | 19 |
| Leeds, U.K. | **14** | 5 | 0 | 0 | **62** |
| France | 9 | 10 | 4 | 7 | 4 |
| U.S. | **2** | 8 | 2 | 6 | 18 |
| Japan | 3 | 0 | 0 | 0 | 0 |

* The *RYR1* mutations listed as column headings have been noted in these percentages of unrelated MHS subjects with pathologic variants in the *RYR1* in the countries noted in the left column. The most frequent *RYR1* mutation is often different from one country to another in Europe. The bold numbers are significantly greater than the others in each column with the exception of the first column. Gly341Arg has been found more often in the U.K. than in the U.S., but there is no significant difference in the incidence of this MH mutation between the other countries noted here.

When the entire coding sequence of *RYR1* was examined in known MHS subjects, variants were found in 70% in North America[16] and Japan[15]. In 50 Italian MHS individuals, the *RYR1* mutation detection rate was 86%[14]. Sequence variants that may be causative of MH have been observed outside the hot spots. Many such variants have been observed in only one MHS family. More *RYR1* variants are reported every year, but variation from normal does not necessarily imply causation of the disease MH. The majority of *RYR1* variants associated with MH are missense. The pathogenecity of these must be carefully evaluated. Strict criteria for proof of causation are available at www.emhg.org as is the list of currently accepted MH-causative mutations. In 2006 there were 28 *RYR1* mutations in this list. This list could be much longer in the future.

If genetic testing of *RYR1* is applied to individuals when there has been no muscle contracture test diagnostic of MH performed in any relatives, the chance of documenting a MH-causative variant is much less. This is in part because 50% or more of patients who experience clinical events that appear to be consistent with MH[17] have muscle that responds normally on the contracture test. These people are not MHS. It is very difficult to diagnose MHS on the basis of clinical signs and nonspecific laboratory tests. When the same portions of *RYR1* are examined, in families who had positive muscle contracture tests, 60% of 129 had *RYR1* variants, whereas in families who had NOT had contracture testing, only 20% of 46 had *RYR1* variants, a strongly significant difference[13]. In other populations[4], the yield of positive results, if genetic testing were the initial test in a family, would be even lower. Seven patients referred for *RYR1* exam due to elevated creatine kinase in the absence of personal or family experience of an acute MH event or positive muscle contracture test did not have *RYR1* mutations[14]. These results support the need to evaluate detailed personal and family history when referring a patient for genetic testing of MHS. This can be done by a MH Diagnostic Biopsy Center or the Genetic Counselor at the Center for Medical Genetics at the University of Pittsburgh Medical Center (D. Steele, #800-454-8155). Better understanding of the effects of *RYR1* variants on daily function and physical performance during stress will be facilitated by accumulation of diagnostic reports with detailed histories in the North American MH Registry (see www.mhreg.org).

## DISCORDANCE

Discordance between MH phenotype, defined by the muscle contracture test, and accepted MH-causative *RYR1* genotypes has been noted[11,13]. Of 363 individuals in 58 well-characterized MHS families, there was diagnostic disagreement, discordance, between muscle contracture result and genotype in 10 subjects[13]. The *RYR1* variants in these families were all functionally tested mutations that could cause clinical MH. In only one of these cases was a negative contracture test found in a patient with a causative MH mutation. This patient had experienced an MH crisis during isoflurane anesthesia. Therefore, this is an instance of a false-negative contracture test. A false-negative contracture test may occur when the muscle is not viable, when it is altered by concomitant medications, or if the concentration of agonists in the contracture test bath was suboptimal. The sensitivity of the contracture test, based on genetic findings in these 196 patients, was 99.5%[13]. This is similar to the sensitivity of the contracture test defined by clinical cases. On the other hand, in nine cases, a patient with MHS diagnosed by muscle contracture testing did not have the familial causative mutation. It is possible that contracture tests have false-positive results because the test is defined with a low threshold so that no individual who might develop MH is not recognized. It is also possible that a second MH-causative mutation that was not detected in other relatives was present in the discordant individual with muscle contracture test results diagnostic of MHS.

In some cases, discordance has been resolved by redefining the cut-off values for the contracture test results[18,19]. This is a reasonable approach if the family is large enough to evaluate linkage with MHS because the definition of positive contracture threshold was based on a large population of subjects. This population-based definition is then applied to a diverse group of unrelated individuals to diagnose MHS after a suspicious clinical event. The threshold for a positive contracture test result was deliberately set at a low level so that the test would not fail to recognize any MHS individual. Therefore, specificity of 85% was accepted as appropriate for the CHCT and as many as 15% of positive CHCT results may be false positives[20]. The sensitivity and specificity of the IVCT are the subject of ongoing study[21,22]. The best statistical analysis for a large population may be inadequate for the analysis of every family[23]. For example, when the population-based definitions of positive IVCT were applied to a large Maori family in which 130 people had been diagnosed by IVCT and 5 had experienced MH episodes; 22 individuals were discordant. These 22 people had MHS diagnosis based on IVCT and they did not have the familial mutation. When more stringent definitions were required to produce an MHS diagnosis from IVCT results, fewer individuals were discordant. When MHS was diagnosed only at an IVCT threshold of 1.2 g contracture in the presence of 2.0 m*M* caffeine and 1.8 g tension in the presence of 2% halothane, there was no discordance between diagnosis by IVCT and by genotype in this family[19]. Unfortunately, most families do not include such a large number of well-studied individuals. Therefore, it is not usually possible to produce family-specific definitions of contracture test diagnostic limits.

When the familial *RYR1* mutation is not identified in an individual with positive contracture test results, the possibility must be considered that a different MH-causative mutation exists in that individual. Two MH-causative *RYR1* mutations have been observed in individuals in several unrelated European families[6], in North America, and in Japan. A family with both an *RYR1* MH-causative mutation and a mutation in the gene encoding another putative MH locus was described in France[6].

## OTHER LOCI POSSIBLY ASSOCIATED WITH MH

For many MHS families, linkage to MHS1 could not be demonstrated[24]. As discussed above, this may be due in part to the nature of the contracture test[23]. Linkage analysis in a few families demonstrated other loci, 17q21-24[25], 7q21-24[26], 3q13[27], 1q32[28], and 5p[29], associated with MHS. MHS2 thorough MHS6, respectively, are the designations of these loci. Because MH is an abnormality of excitation-contraction coupling, the gene encoding the protein that forms the calcium-release channel of the muscle sarcoplasmic reticulum, *RYR1*, and the genes encoding subunits of the adjacent voltage-gated channel, the dihydropyridine receptor (DHPR), are logical candidates for linkage with MH. The DHPR is a heterotetramer encoded by four genes. The *CACNA1S* or *CACNL1A3*, encoding the $\alpha$1-subunit of the DHPR, is at 1q32[28]. Polymorphisms and potential mutations have been identified in this gene[30]. In one family, the Arg528His amino acid substitution in the α1-subunit of the DHPR encoded by *CACNA1S* produced a severe form of hypokalemic periodic paralysis. This mutation was present in the young man in that family who died from anesthetic complications resembling MH[31]. In a different large French family, a mutation in *CACNA1S* was linked to MHS[32], but hypokalemic periodic paralysis can also occur independently of MHS diagnosed by IVCT[33]. The *CACNLB1* encoding the β-subunit and the *CACNLG* encoding the γ-subunit are both located in 17q11.2-q24[34,35] and the *CACNLA2* encoding the α2/δ-subunit of the DHPR is in 7q11.23-q21.1. Thus, the genes encoding the subunits of the DHPR are candidate genes for MH loci 2, 3, and 5, but these genes have not been linked to MHS in families. No candidate genes have been determined for MH loci 4 and 6[36].

Interaction between several genes may influence the MH phenotype within one family[36]. Candidate loci were examined in 131 families from different areas in Europe in which MHS had been diagnosed by means of IVCT. *RYR1* was associated with MHS as were loci on chromosomes 1, 5, and 7 in these families. When the 100 families in which there was a high probability of linkage between MHS and *RYR1* were considered separately, there was still an association between MHS and loci on chromosomes 5 and 7. In the families for which there was no evidence of linkage between MHS and *RYR1*, there was an association of MHS with a locus on chromosome 7.

Many mutations in genes encoding subunits of muscle voltage-gated ion channels have been described. Different clinical disorders may be related to variants in one gene and one clinical disorder can be associated with variants of several genes. For example, hyperkalemic periodic paralysis, potassium aggravated myotonia, paramyotonia congenita, and hypokalemic periodic paralysis type 2 are associated with mutations in the human skeletal muscle sodium channel. The gene encoding the adult muscle sodium channel (*SCN4A*) is located on chromosome 17q23.1-q25.3. *SCN4A* has been proposed as a MH locus in a few pedigrees in the western hemisphere[25,37,38,39]. In one of these pedigrees, reports of MH were based primarily on clinical events. Stiffness followed administration of succinylcholine in all three patients diagnosed as MHS. Acidosis, fever, and rhabdomyolysis were variable. The one CHCT performed was prior to standardization of this test. Weakness was a frequent complaint after general anesthesia[39]. Furthermore, no European families have been found to have *SCN4A* linked with MHS[40,41]. Although people with either hyperkalemic or hypokalemic periodic paralysis may experience significant anesthetic complications similar to those associated with mutations in *RYR1*, there are also important differences between diagnostic findings and potential treatments for people with disease related to *SCN4A* vs. *RYR1*. Furthermore, a rare disease such as hypokalemic periodic paralysis may be associated with mutations in *CACNA1S* (type one hypokalemic periodic paralysis), *SCN4A* (type two hypokalemic periodic paralysis), or *KCNE3* on chromosome 11q13-q14 encoding MinK-related peptide 2, which is part of a skeletal muscle potassium channel[33].

It is not clear that dysfunction of the sodium and potassium channels will necessarily result in excessive calcium release through the RYR1 or some other abnormality of intracellular calcium in muscle. There is much evidence to support the claim that stiffness associated with MH and with myotonias are not the same disease[42], but it is to be expected that when myotonia is a clinical problem, excessive stiffness with subsequent increased metabolism and possible muscle injury will follow administration of succinylcholine, the only depolarizing neuromuscular blocker in clinical use.

## MYOPATHIES AND MH

Central core disease (CCD) is an uncommon myopathy described in 1956 as the first congenital myopathy with structural changes in muscle fibers[43]. The diagnosis of CCD rests on histopathology. There is predominance of type one fibers containing centrally located areas lacking mitochondria and variable disintegration of the contractile apparatus in this core region[44]. CCD also has variable clinical manifestations. CCD has been associated with variants in the *RYR1*[45,46,47]. Some *RYR1* mutations produce both MH and CCD. Myotubes expressing RYR1 channels associated with both MH and CCD exhibited voltage-gated calcium release at more negative potentials and had a higher incidence of spontaneous calcium oscillations than did myotubes containing normal RYR1[46]. The same was true of some RYR1 variants associated with MH and not CCD. The RYR1s associated with both MH and CCD also had lower maximal voltage-gated calcium release and calcium oscillations occurred with longer duration or greater frequency than those associated only with MH[48]. These laboratory observations suggest that CCD associated with variants of *RYR1* can be expected to be MHS, but the function of all RYR1 variants associated only with CCD, in the absence of documented MH, have not been examined, and the functional properties of RYR1 variants may be quite different[46]. There are some *RYR1* mutations associated with CCD that do not cause MH[49]. As more of the *RYR1* is examined in patients with CCD, *RYR1* variants are found in a larger percentage of patients[50].

Usually CCD is autosomal dominant. In contrast, multiminicore disease (MmD) is an autosomal recessive congenital myopathy. In common with CCD, the cores in MmD lack mitochondria, but they differ in that they do not extend the length of the muscle fiber[44]. Mutations in selenoprotein N (*SEPN1*) have been the most frequent finding in MmD[51], but other cases of MmD have been found to have variants in the *RYR1*[52]. CCD may initially present as MmD. Nemaline rods have been found in some CCD patients[53], although nemaline rod myopathy has been described as a disease entity of the muscle cytoskeletion different from CCD. There are other examples of clinical overlap between these myopathies, associated with variants of RYR1[54,55].

The majority of classic MH cases are associated with variants of *RYR1*, but there are clinical situations in which other types of pathophysiology are associated with signs and symptoms resembling classic MH. Researchers focused on describing the genetics of the MH syndrome argue that only abnormalities of the excitation-contraction coupling mechanism in muscle can be considered to be MH. However, the clinician has to care for patients with diverse, often undiagnosed, muscle diseases. Which patients should be treated as if they are MHS? Some anesthesiologists may argue that all patients suspected of muscular disease should be treated as if they could develop MH, but it is not clear that avoidance of all inhalation anesthetics is always in the best interest of the patient with muscle disease who may NOT be MHS. It may be difficult to determine objectively if MHS is to be expected in a particular myopathy because few myopathic patients undergo CHCT and because of the clinical overlap between some of the rare myopathies.

There are myopathic patients in whom MH-like reactions have occurred during anesthesia in which the primary abnormality is a defect in structural elements of the sarcolemma. For example, a child with merosin (laminin $\alpha$-2) deficiency experienced increased heart rate, increased carbon dioxide production, and increased temperature during total intravenous anesthesia in the absence of MH-triggering agents. These signs of increased metabolism resolved after dantrolene was given[56]. Merosin is a structural element in the subsarcolemmal space. Dystrophin is another structural protein in the subsarcolemma. There are many cases of patients with Duchenne muscular dystrophy, due to absence of dystrophin, (DMD) who experienced anesthetic complications during or after exposure to potent inhalation agents[57]. Although these events were often described as MH episodes, they differ in some respects. Some patients with DMD have undergone muscle contracture testing with positive results. In contrast, negative CHCT results have been obtained in young boys with dystrophinopathy and in animal models of DMD[58]. Increased intracellular calcium is thought to be part of the pathogenesis of DMD, but why this occurs is not known[59]. There are no significant differences in the transverse tubular system or the voltage dependence of calcium release from the sarcoplasmic reticulum (SR) in DMD and normal muscle fibers, but the amount of calcium released from the SR in DMD is less than normal[60]. Calcium binding proteins in the SR of DMD muscle may be much less than normal[61]. There may be different sources of calcium influx in DMD muscle than in normal[62]. So although increased intracellular calcium may be a common feature in some muscular dystrophies and classic MH, the causes of this differ and the associated genetic loci differ.
